# Partnering With Lived Experience Experts to Co‐Design and Co‐Facilitate Trauma‐Informed Practice Training for Health Professionals: Evaluation Findings

**DOI:** 10.1111/hex.70883

**Published:** 2026-09-30

**Authors:** Sarah Ciftci, Susan Heward‐Belle, Nicole Pryor, Betty Luu, Hannah Mayjor, Portia Freeman, Fiona Rees, Renee Lovell

**Affiliations:** ^1^ Sydney School of Education and Social Work University of Sydney Sydney New South Wales Australia; ^2^ Community Health Services Sydney Local Health District Sydney New South Wales Australia; ^3^ Transforming Early Education and Child Health Research Centre Western Sydney University Sydney New South Wales Australia

**Keywords:** co‐design, co‐facilitation, evaluation, lived experience, professional learning, training, trauma‐informed practice

## Abstract

**Background:**

There is growing demand for health professionals to be upskilled in trauma‐informed practice to provide dignity‐based healthcare responses to clients with lived experience of child sexual abuse and other adverse childhood experiences. Traditionally, such training has been developed and delivered by clinical experts or academics with specialised training, excluding those with lived experience expertise. Limited research has explored the involvement of lived experience experts in professional learning.

**Methods:**

This study evaluated the quality and impact of a trauma‐informed practice training package for community health, mental health and drug health professionals that was co‐designed and co‐facilitated by women with lived experience of child sexual abuse. A feedback questionnaire utlising a single‐assessment retrospective pre‐post design coupled with open‐ended questions was administered and qualitative interviews were conducted via a purposive sampling approach. In total, 97 survey responses and 10 qualitative interviews were included in the study. Descriptive and inferential statistics were generated to measure changes to knowledge and confidence as a result of the training and to examine which aspects of the training were most valuable. Thematic analysis of data was conducted to explore how lived experience co‐facilitation supported learning.

**Results:**

There was an increase in both the proportion and mean scores of participants who reported feeling confident in understanding trauma informed practice immediately after the training. Paired sample *t*‐tests indicate these increases were statistically significant (*p* < 0.001). Hearing directly from a trainer with lived experience was the most valued aspect of the training and was given the highest rating of ‘excellent’ by the highest proportion of participants (85%) compared to all other aspects of the training. Five key qualitative themes were identified, demonstrating how: (1) the depth and richness of lived experience perspective supported learning, (2) humanised trauma‐informed practice beyond theory, (3) encouraged engagement through openness, (4) facilitated the translation of learning into practice, and (5) informed improvements to workplace practices.

**Conclusion:**

Future trauma‐informed practice training for healthcare professionals can utilise lessons learned in this study to enact participatory co‐design approaches and leverage the strengths of lived experience expertise for professional learning.

**Patient and Public Contribution:**

Three women with lived experience of child sexual abuse were employed in the study as lived experience experts alongside the academic researchers. They provided input into the design and development of the training content and co‐facilitated each training session alongside a clinical expert. They reviewed this manuscript and are co‐authors. The research and training were also informed by co‐design listening sessions with 44 people with lived experience of child sexual abuse and an Advisory Group of seven young adults with lived experience of child sexual abuse.

## 1 Introduction

### 1.1 Health Impacts of Child Sexual Abuse

Child sexual abuse (CSA) is a common and egregious human rights violation globally and within Australia where this study was conducted. CSA encompasses acts that involve or expose a child to sexual processes beyond their developmental understanding or contrary to accepted community standards and law [1]. Globally, approximately one in five girls and women and one in seven boys and men experience sexualized violence and abuse during childhood [2]. In Australia, a landmark prevalence study reported that one in three girls and one in seven boys are subjected to CSA [3]. Victim‐survivors are at substantially elevated risk of mental and physical health harms across the lifespan, including suicide and suicidal ideation, self‐harm, substance dependence and chronic physical conditions [3]. It is noteworthy however, that not all individuals subjected to sexualized abuse in childhood go on to develop persistent, long‐term symptoms.

It is therefore important for health professionals to operationalise trauma‐informed approaches to understand the healthcare needs of those who have been subjected to CSA. In Australia, the *Royal Commission into Institutionalised Responses to Child Sexual Abuse* (2017) found that many victim‐survivors experienced the health system as re‐traumatising, particularly mental health services. They reported inconsistent access to trauma‐specific care and clinicians who lacked understanding of the long‐term impacts of childhood sexual abuse [4]. This is consistent with academic literature which draws attention to how systemic shortcomings often compound harm [5]. Despite localised efforts in implementing trauma‐informed practice (TIP) within health systems, overall health systems have been widely described as undermining the dignity of system users, with those who access health systems frequently reporting feeling marginalised in their encounters with health professionals [6, 7]. High levels of disempowerment, stigmatisation and coercion within mental health settings have been widely documented [8, 9]. Accordingly, the Royal Commission recommended that health services adopt trauma‐informed and victim‐survivor‐centred approaches across all levels of care (recommendation 9.2) and that the health workforce receive mandatory education and training on the impacts of CSA and TIP (recommendations 9.6, 9.7).

### 1.2 TIP in Healthcare

TIP in healthcare recognises the pervasive impact of trauma on physical, psychological, and social wellbeing and how it can shape engagement with health services, integrating this understanding into all aspects of care delivery. Rather than focusing solely on symptoms or diagnoses, TIP reframes clinical encounters to prioritise safety, trustworthiness, choice, collaboration, and empowerment [10] whilst attending to a person's culture, history and gender. As such, TIP shifts the focus from pathologizing behaviours (‘What is wrong with you?’) to understanding a person's life experiences (‘What happened to you?’). TIP implementation encompasses both organisational and clinical practices. Examples of organisational practices include engaging service users in planning and service design, TIP training and workforce development, and policies and strategies for preventing secondary traumatic stress in staff. Examples of clinical practices include screening and assessing for trauma, involving patients in the treatment process and providing or referring to trauma‐informed treatment services [11]. Evidence suggests that trauma‐informed approaches can improve service engagement, therapeutic relationships, consumer satisfaction, and staff wellbeing, particularly in mental health, drug health and community healthcare settings [12, 13]. As a result, TIP is increasingly viewed as a foundational component of quality and ethical healthcare creating substantial demand for workforce upskilling [14].

Evaluations of workforce TIP training consistently demonstrate benefits, including improved trauma knowledge, increased confidence in responding to disclosures, and greater awareness of referral pathways [15, 16, 17, 18, 19]. There are, however, commonalities and variations in TIP training curricula and delivery methods [20] and very few studies report co‐design and co‐facilitation of TIP training with service users in professional development contexts [17]. The few evaluations of victim‐survivor co‐facilitated training that exist in other contexts, including training for professionals involved in the Independent Inquiry into Child Sexual Abuse in England and Wales and for humanitarian aid workers, found that participants reported significant benefits translated into victim‐survivor‐centred practice learning [21, 22].

### 1.3 Involvement of Lived Experience Experts (LEEs) in Health Education and Training

There has been a growing movement in medicine, nursing, allied health and mental health toward consumer involvement in health profession education, driven by evidence that such inclusion better equips future healthcare professionals to engage compassionately with people experiencing mental distress [23, 24, 25, 26, 27, 28, 29]. This shift also responds to the need to recognise consumers as credible knowledge holders to advance epistemic justice [30] and centre dignity in professional practice. Mental health consumers involved in health professional education have been referred to as ‘consumer educators’, ‘Experts by Experience (EbE)’, ‘Lived Experience Experts’ or ‘Lived Experience Educators’. In this paper, people with lived experience of CSA who contributed to training design and delivery are referred to as ‘Lived Experience Experts (LEEs)’.

Research demonstrates that learning from LEEs reduces stigma, increases empathy, and models collaborative practice [23, 31, 32]. University students who participated in co‐designed and co‐delivered recovery‐oriented practice workshops with LEEs showed statistically significant improvements in their recovery knowledge and capabilities, highlighting that meaningful LEE involvement enhances learning outcomes and promotes person‐centred practice [26].

This study sought to understand the value of LEE involvement in TIP professional learning and contribute to the limited evidence base in this context. To our knowledge, this is the first study to evaluate TIP training co‐designed and co‐facilitated by victim‐survivors of CSA delivered to both acute‐ and community‐based health professionals.

## 2 Methods

A mixed method evaluative study was conducted in Sydney, Australia to explore the impact of a lived experience co‐designed and co‐facilitated TIP training package. Ethics approval was granted for the study by the University of Sydney Human Research Ethics Committee (Approval number: 2023/623) and Sydney Local Health District Ethics Review Committee (Approval number: X24‐0418). This paper reports findings in relation to two research questions: (1) *what was the impact of the training on the knowledge and confidence of participants?* and (2) *how did lived experience co‐facilitation contribute to the learning experience of participants?*

### 2.1 Research Context

The TIP training was developed and delivered in partnership with Sydney Local Health District, as part of a broader Australian participatory action research (PAR) study called *Dignity by Design: Victim‐Survivor‐led System Redesign*, that aimed to enhance the capacity of policymakers and practitioners to reform service systems that often worsen and sustain harm for victim‐survivors of CSA. Three paid LEEs were employed as co‐researchers on the research team, and a project advisory group comprising seven young adults with lived experience of CSA provided ongoing advice. Qualitative data gathered in Phase One from 44 young adults with lived experience of CSA identified pervasive affronts to dignity in health services, particularly in mental health settings and centred upskilling health professionals as a priority [33].

The TIP training was implemented during Phase Two of the study, building on existing work from Sydney Local Health District's Adult Survivors’ Program (Integrated Services for Adult Survivors of Child Sexual Abuse with Complex Needs), and findings from Phase One of the Dignity by Design study. This was also informed by cultural consultations with Yamurrah Aboriginal Collective. The TIP training was co‐designed through active collaboration [34] between three LEEs, three clinician educators and two researchers over 6 months. Regular co‐design meetings were held to agree content and activities. Mentoring and coaching opportunities, including LEEs co‐facilitating tutorials to tertiary education students occurred to build confidence and skills for training delivery.

### 2.2 Training Components

Staff in Sydney Local Health District were invited by email to a full‐day TIP training, co‐facilitated by a health clinician educator and a LEE, utilising educational presentations, LEE insights, interactive learning activities and provision of educational materials. Care was taken to create a safe physical and emotional environment. Workshop content addressed foundational components of trauma‐informed approaches including: the neurological and epigenetic impacts of trauma; adverse childhood experiences research; trauma responses across the lifespan; trauma‐informed principles; relational healing; the importance of listening; slowing down and being present; and vicarious trauma, self‐awareness and self‐care. The training was delivered using a trauma‐informed approach, with participants seated in a circle and group numbers intentionally limited to promote psychological safety. The venue was selected for its natural light, views of surrounding trees, and access to an outdoor area that was incorporated into activities and breaks. The slide deck presented material in a non‐confrontational manner, drawing on nature‐based imagery to support emotional regulation. Regular comfort breaks were provided, along with mindfulness supports such as breath work, polyvagal exercises, colouring pages and fidget tools. A range of experiential learning activities enabled participants to exercise choice and control in their level of engagement and to meet the needs of different adult learners. The training also modelled trauma‐informed micro‐practices and offered opportunities to develop applied skills through structured role‐play exercises. The training was free to attend, was incorporated into the Sydney Local Health District training calendar, and was attended and endorsed by executive, thus creating an authorising environment. This was considered an essential element of ensuring the training was trauma informed. Those who completed the course had it recorded in their staff learning profile.

### 2.3 Training Implementation and Data Collection

One hundred and six professionals completed the training across seven sessions held between May and October 2025. A purposive sampling approach was used: facilitators presented study information at the beginning of each session, inviting participants to complete an optional brief anonymous survey and follow‐up interview. No incentives were offered.

The anonymous survey comprised both quantitative and qualitative elements and was purpose‐designed to align with training content. The quantitative component utilised a single assessment retrospective pre‐post design (SARPPD), which assessed pre‐ and post‐training confidence and knowledge in a single post‐training self‐report, eliminating the need for multiple timepoints which reduced time burdens for participants [35]. This design also addresses response‐shift bias, wherein participants’ assessments of their pre‐training skill levels are often recalibrated following program completion [36]. Participants were asked to self‐report their level of knowledge across six anticipated learning outcomes related to TIP. They were also asked to rate four aspects of the training delivery including hearing directly from a trainer with lived experience as well as to provide a rating of the training overall. Five‐point scales were used.

Participants interested in a follow‐up interview registered contact details and provided consent via a QR code linked to a Qualtrics form. Ten interviews were conducted, ranging from 30 to 60 min, held online via Microsoft Teams, and facilitated by a researcher with no pre‐existing relationships with participants. All interviews were audio‐recorded and transcribed verbatim. Interviews utilised the discussion guide developed by the researchers and informed by co‐design listening sessions with 44 victim‐survivors. Open‐ended questions were asked regarding the quality and utility of the training and the impact of hearing directly from a victim‐survivor.

### 2.4 Sample

Of 106 participants, 100 completed the survey; 97 consented for inclusion (91.5% response rate). No personal or demographic information were collected via the survey to preserve anonymity; however, basic information was collected via sign‐in sheets including department (98.2%) and in some cases role (57%). As shown in Table 1, participants worked in a range of Sydney Local Health District departments most commonly Community Health Services (30.2%), Mental Health Services (24.5%) and Drug Health Services (18.9%).

**Table 1:** Training participant details.

Training participant details	No. participants (%)
Department	
Community Health	32 (30.2)
Mental Health (including hospital and community‐based services)	26 (24.5)
Drug Health (including hospital and community‐based services)	20 (18.9)
Hospital setting (unspecified)	12 (11.3)
Workforce education and training	6 (5.7)
Aged health, chronic care and rehabilitation	5 (4.6)
Dental health	1 (0.94)
Safe work	1 (0.94)
Cultural support	1 (0.94)
Unknown	2 (1.8)
Role	
Nurse	34 (32)
Social worker	9 (8.5)
Allied health worker (e.g., occupational therapist, nutritionist)	7 (6.6)
Psychologist or counsellor	4 (3.8)
Team support	4 (3.8)
Leadership (e.g., director, clinical governance, program manager)	3 (2.9)
Doctor	2 (1.9)
Unknown	43 (40.5)
Total	106 (100)

Ten professionals participated in follow‐up interviews between 4 weeks to 4 months post‐training (x̄ 4 weeks). As shown in Table 2, most (*n* = 9) were female and half (*n* = 5) worked in Community Health Services. Interview participants held a diverse range of roles with social worker being the most common (*n* = 4).

**Table 2:** Interview participant details.

Interview participant details	No. participants
Gender	
Female	9
Male	1
Department	
Community health	5
Mental health (hospital setting)	2
Aged health, chronic care and rehabilitation	2
Drug health (including hospital and community‐based services)	1
Role	
Social worker	4
Nurse	2
Speech pathologist	1
Psychologist	1
Doctor	1
Health promotion officer	1
Total	10

### 2.5 Data Analysis

Quantitative data were summarised using descriptive and inferential statistics, standard deviations and frequencies (percentages) were generated and paired sample *t*‐tests were conducted to test for significance. Quantitative results are described in more detail below. Qualitative data including open‐ended survey responses and interview transcripts using reflexive thematic analysis following Braun and Clarke's six‐phase iterative process [37]. This involved immersive reading and re‐reading, generating initial codes within and across transcripts, collating codes into themes and developing and refining themes to produce a detailed analytic narrative. Five themes were generated. NVivo15 was used to assist with data storage and management.

## 3 Results

### 3.1 Quantitative Results

#### 3.1.1 Changes in Knowledge and Confidence

Participants were asked to provide their ratings on a scale of 1 to 5—with 1 being ‘not confident at all’, 3 being ‘neither’ and 5 being ‘very confident’—in relation to a series of six statements exploring changes to their knowledge and confidence of trauma informed practice before and after the training. Increases in the percentage of participants reporting confidence and mean scores from before to after the training indicate a positive shift in knowledge and confidence for all participants. As summarised in Table 3, the percentage of participants who reported being ‘fairly confident’ or ‘very confident’ increased from before the training to after the training across all statements. Likewise, mean ratings increased from before the training to after the training across all statements, with paired sample *t*‐tests revealing each increase was statistically significant. The most pronounced changes in knowledge and confidence were in relation to the statements: ‘I understand what it means to be Trauma Informed and the theory behind Trauma Informed Practice’, ‘I understand how to apply the principles of Trauma Informed Practice (safety, trust, choice, collaboration, empowerment, culture) in my work’ and ‘I am confident I can provide Trauma Informed responses to clients’. Participants reported the lowest levels of confidence before the training across these three statements and the most substantial shifts to higher confidence levels.

**Table 3:** Knowledge and confidence before and after training.

Statement	Percentage % ‘fairly confident’ or ‘very confident’ before training	Percentage % ‘fairly confident’ or ‘very confident’ after training	Mean (SD) rating before training	Mean (SD) rating after training	Paired sample *t*‐test *t*(93) = *p* value
I understand how widespread Trauma is and how it impacts health and wellbeing	50	100	3.34 (SD = 1.01)	4.63 (SD = 0.49)	*t*(93) = −12.81, *p* < 0.001
I understand what it means to be Trauma Informed and the theory behind Trauma Informed Practice	37	99	3.05 (SD = 1.01)	4.56 (SD = 0.52)	*t*(93) = −13.92, *p* < 0.001
I understand how to apply the principles of Trauma Informed Practice (safety, trust, choice, collaboration, empowerment, culture) in my work.	37	99	3.02 (SD = 1.07)	4.46 (SD = 0.52)	*t*(93) = −13.49, *p* < 0.001
I am able to recognise trauma symptoms for clients, colleagues and myself	68	98	3.51 (SD = 0.93)	4.38 (SD = 0.53)	*t*(51) = −7.39, *p* < 0.001
I am confident I can provide Trauma Informed responses to clients	39	95	3.11 (SD = 1.08)	4.41 (SD = 0.59)	*t*(92) = −13.33, *p* < 0.001
I understand what vicarious trauma (indirect trauma) is and the self‐care strategies and tools I can use to mitigate its impact	50	96	3.24 (SD = 1.15)	4.53 (SD = 0.58)	*t*(93) = −12.06, *p* < 0.001

#### 3.1.2 Quality of Training

Participants gave highly positive overall ratings of the trauma informed practice training. As shown in Table 4, 69% rated the training as *excellent*, and a further 22% as *very good*; no participants rated it as *fair* or *poor*. Hearing directly from a trainer with lived experience was the most highly rated aspect of the training (85% excellent), surpassing trainer knowledge (75% excellent), training ability to relate material to participants’ work environment (75% excellent), and usefulness of resources (56% excellent) as shown in Table 5.

**Table 4:** Rating of training overall.

Question	Poor	Fair	Good	Very good	Excellent
How do you rate this training overall out of 5?	0%	0%	9%	22%	69%

**Table 5:** Rating aspects of training.

Statement	Poor (%)	Fair (%)	Good (%)	Very good (%)	Excellent (%)
The level of knowledge the trainers had about Trauma Informed Practice	0	0	3	22	75
Ability of the trainers to relate material to your work environment	0	2	7	15	75
How useful/relevant the training resources (e.g., handouts) are	0	2	9	33	56
Hearing directly from a trainer with lived experience	0	0	3	12	85

Hearing directly from a trainer with lived experience was the most valued aspect of the training as shown in Table 5. Participants were asked to rate four aspects of the training: (1) knowledge levels of trainers, (2) ability of trainers to relate to work environment (3) usefulness and relevance of training resources, and (4) hearing directly from a trainer with lived experience. Of the four aspects of the training, hearing directly from a person with lived experience was given the highest rating of ‘excellent’ by the highest proportion of participants (85%) compared to all other aspects of the training.

### 3.2 Qualitative Results

The following five key themes were generated from an analysis of the qualitative data.

#### 3.2.1 Theme 1: Learning From the Depth and Richness of Lived Experiences

Participants spoke highly of the profound impact of hearing directly from a LEE, positioning this as the most valuable training component. They emphasised the lasting influence of LEE contributions far beyond the training context.

That was the powerful part. To hear her story and the way she articulated it was, yeah, [I] haven't been able to stop thinking about it all, to be honest.

Interviewee 2

The thing that stood out for me was the power of the lived experience and that was what, whenever I talked to others who've been, it was the same thing… [you're] dealing with health clinicians who are quite cut off and their capacity to hold people in mind and see them outside of their disease, their symptoms and the medical model is a disaster… So, I really valued, and I know others did, hearing the lived experience and the person saying, this is what it's like for me to travel in this system rather than the othering that happens.

Interviewee 10

The depth of LEE contributions was identified as enhancing the quality of discussions throughout the day and adding a dimension that professional‐only delivery could not provide.

It was not new information because I've been around for a long time. But the way it was presented like that with the lived experience, it just added a whole new layer, new dimension to the message.

Interviewee 3

#### 3.2.2 Theme 2: Humanising TIP Beyond Theory

LEE co‐facilitation was reported to bridge the divide between theory and practice, keeping focus on the human experience of clients rather than clinical expertise or the biomedical model.

Having the person with lived experience really helped to give insights that have never occurred to myself… It's a really helpful reminder [of] why we're sitting in the room. It makes it far less theoretical and more practical.

Interviewee 1

I think sometimes when you sit in clinical practice days you can get, I mean it's happened to me before, you can get kind of caught up in the theory and what the evidence says. But actually hearing about what it's like from an individual who has been on the receiving end of that or who has experience I think really keeps the whole day grounded in oh, yeah, there's a human experience to each individual person here which I think is really helpful.

Interviewee 8

It's quite powerful to hear those reflections, but also it's theory in practice as well … it's real, it's not something that we've read. It was experienced from a person sitting in the room with us.

Interviewee 9

Participants also highlighted how LEE contributions animated statistics presented with during the training, making the data meaningful and urgent.

You talk about all the statistics… but someone's sitting there going, ‘that was me. I've been through this and look at what we can do to help this not happen to someone else.’…It grounds the session, it brings the focus in that we are talking about real people and it's not an abstract kind of thing, which I think is very important at this point.

Interviewee 2

I found every aspect of the training really helpful, but of course, was particularly keen on the living experience and how that person was given a voice and able to provide examples and really move away from stats because statistics help a bit, but it's people's stories that are always the answer to everything.

Interviewee 10

#### 3.2.3 Theme 3: Encouraging Participation and Engagement Through Openness

The openness and honesty of the LEE co‐facilitator was identified as creating conditions in which participants felt able to ask questions and engage in difficult discussions throughout the day.

I think it also made people more willing to engage in some of the other conversations around it. Around, ‘what was it like?’ and ‘is it really that bad?’…she was incredibly open.

Interviewee 2

I was just captivated by her just the way she spoke so openly and honestly throughout the day… I thought she answered that question really well. She was amazing in what she shared and how she responded to people's questions and put the material into context, real life.

Interviewee 3

#### 3.2.4 Theme 4: Putting Strategies Learned Into Practice

Several participants described changes to their practice following the training, including slowing down to listen, acknowledging traumatic experiences, attending to their non‐verbal cues, enquiring about support needs, and making appropriate referrals or engaging in advocacy.

I probably did apply it to the mum [of a CSA survivor] I saw last week. Because she of course burst into tears just telling me…I was much more calm just sitting there and listening, not trying to diminish but you know acknowledging it but then some of the strategies from the training were, ‘Do you feel like this is still something that you want to get support for?’ Even though this was something you know 5 years in the past, not taking it for granted that they'd actually had closure that event yet.

Interviewee 1

Just listening without thinking you know the answer already, and also if someone wants to talk to you, just like talk to them … as soon as someone walks in, just stand up straight away like no phone… that person just has my full attention if they want it. Some people don't, and that was another really big thing I took away is like some people can be freaked out by eye contact. Some people don't want to talk to you. So, I think I've become way more attuned to just looking for those cues and on the whole I think the thing for me has been just been being as open as possible.

Interviewee 5

#### 3.2.5 Theme 5: Using Learnings to Improve Workplace Practices

Participants described how they planned to, or had already begun to, advocate for improved TIPs within their teams and services, including challenging colleagues’ non‐trauma‐informed language and behaviours.

There's a couple of conversations I'm going to have with some staff members who are currently on leave. A couple things that they've done that I don't think is the best trauma informed practice.

Interviewee 2

[I'm] taking on board how [lived experience facilitator] approached it. Telling people they're doing the wrong thing is not the way to get them to change their behaviour. So, what are some ways that I can slowly influence that over time that feel more aligned with my values and how I think we should be talking about people that have experienced these things? So [my practice change] is probably more on that side of things in terms of working with colleagues… I've decided to start with, I really hate the way [Borderline Personality Disorder] gets thrown around as a label, often to describe people's responses to trauma, and I know why it's done…but I can choose not to do that and I can model different language and behaviour instead. And I'm trying to be really consistent about that…There is one consultant in particular who comes to mind who on one or two occasions I have said ‘I think if the patient heard you talk about them like that, I don't think they'd like it. Maybe we could say it a different way’ who has been like ‘Oh, yeah, that's a good point’. [They've] attempted in my presence, at least, to change what they're doing.

Interviewee 8

Participants discussed how they planned to advocate for improved TIPs in their services and disseminate their learnings through team meetings and conversations with colleagues. One participant highlighted how the training had prompted sustained advocacy for co‐design with LEEs to be embedded across all aspects of service design and delivery.

I am always asking now, is it co‐design and who's the people you're working with? And also, have you got a lived experience on your working group, on what you're doing? And people don't necessarily welcome it or like it…but how can I communicate to others in those other settings, hey, why aren't you thinking of this?…Why isn't that central in your training?…And to remind me of my own interactions and we're doing an adolescent brochure now. So, it's now always front of mind again. Okay, which young people will we ask about this resource? Who can we talk to?

Interviewee 10

## 4 Discussion

The study highlights the transformative potential of embedding lived experience expertise in the design and delivery of TIP training to enhance workforce capability in health settings. It contributes to the small but growing body of literature evidencing the value of partnering with LEEs to develop workforce knowledge and skills in delivering trauma‐informed and dignity‐centred healthcare [26, 38, 39].

The results support conceptualising LEEs as epistemic partners with tangible benefits for professional learning making a case for prioritising such partnership in professional development activities. Returning to the concept of epistemic injustice, Fricker [30] identifies two primary forms: testimonial injustice (dismissal of testimony due to negative identity‐based bias) and hermeneutical injustice (when marginalised groups are excluded from accessing interpretive resources such as concepts, ideas and narratives). Fricker [40] and others [41, 42] have since expanded and named various forms that epistemic injustice may take in specific domains of knowledge making. One such form that is particularly salient to this study is participant‐based injustice, which shifts attention from credibility and interpretive gaps to who is enabled or prevented from participating in epistemic practices at all. Participant‐based injustice foregrounds exclusion from the processes through which knowledge is produced, shared, and validated. It highlights that injustice does not only occur when individuals are disbelieved (testimonial injustice) or lack interpretive resources (hermeneutical injustice), but also when they are systematically marginalised as contributors to knowledge practices in the first place.

Victim‐survivors have historically been marginalized as witnesses but not as contributors to knowledge practices that shape the services meant to support them. In co‐designing and co‐facilitating this training, we positioned their knowledge as central, distinct and valuable, and this positioning shaped how their contributions were received. Participants emphasised how LEE contributions humanised training content and challenged entrenched biomedical paradigms that prioritise symptom management over relational safety and dignity. In our view, partnering with LEEs in TIP training is itself an enactment of the principles of TIP: safety, trustworthiness, choice, collaboration, and empowerment, and an acknowledgement of culture, history and gender, that we aim to instil in health practice.

Meaningful co‐production, in which LEEs move beyond tokenistic consultation toward genuine partnership and shared decision‐making, is associated with more relevant and effective training and with empowering participants to become resourceful practitioners [43, 44]. However, co‐design processes can place considerable emotional demands on LEEs; participation in professional healthcare settings carries real risks of re‐traumatisation and disillusionment if adequate support structures are absent [43]. Organisations must therefore establish clear governance frameworks, provide ongoing psychological support and supervision, ensure fair remuneration, and clarify role boundaries before LEEs are engaged [45, 46].

There remains a tension between professional credentialing cultures and lived experience expertise whereby some professional stakeholders do not equally value experiential knowledge and formally credentialled knowledge. This can undermine LEE authority in training spaces [47, 48]. Research indicates that the presence of victim‐survivor facilitators can produce attitudinal shifts in learners that didactic professional‐only delivery does not achieve, including disrupting implicit biases and building genuine empathy. However, this depends on LEEs being positioned as credible experts rather than as ‘illustrative cases.’ [21, 22] Consistent with a reflexive approach [48], facilitators and course designers should continually examine their own positionality and the power dynamics embedded in whose knowledge is centred, whose is marginalised, and how training content may inadvertently perpetuate institutional responses that have historically failed victim‐survivors.

TIP implementation research demonstrates that, to facilitate high attendance, and sustainable change within health services, it is essential to ensure executive understand how the TIP is aligned with organisational goals, and that training is funded and actively promoted by both executive and front‐line managers [14]. This includes making time for clinicians to attend training, as well as supporting implementation of changes towards increasing the use of TIP beyond a clinicians’ personal practice, following their attendance.

## 5 Limitations

The instrument used for measuring changes to knowledge and confidence was purpose‐designed and not validated, limiting cross‐study comparisons. Generalisability is constrained by single‐site recruitment in non‐research conditions. No demographic data or prior TIP training history were collected from survey respondents, limiting exploration of how these variables may have influenced outcomes. No comparison group was utilised, so causality between LEE co‐design, co‐facilitation and outcomes cannot be established. No long‐term follow‐up was conducted to examine sustainability of outcomes. The training was designed specifically with women CSA victim‐survivors and may need to be adapted for victim‐survivors of other genders or backgrounds.

## 6 Conclusion

This study evaluated the quality and impact of TIP training for health professionals co‐designed and co‐facilitated by women with lived experience of CSA in partnership with health clinician educators. Quantitative findings demonstrate statistically significant improvements in professionals’ confidence and knowledge across all domains. Qualitative findings reveal that participants value LEE integration as the most impactful component of the training, describing it as a driver of learning that transcended theoretical content and prompted tangible practice change. Future TIP training should ensure genuine partnership with LEEs from design to delivery, and an authorising environment for participating in training during clinical hours to realise similar gains and embody the principles of trauma‐informed care in the learning environment itself.

## Author Contributions

**Sarah Ciftci:** conceptualization, investigation, methodology, writing – original draft, project administration, formal analysis, visualization, data curation, resources. **Susan Heward‐Belle:** conceptualization, investigation, funding acquisition, writing – review and editing, supervision, methodology, project administration, resources. **Nicole Pryor:** methodology, writing – review and editing, conceptualization, data curation, project administration, resources. **Betty Luu:** formal analysis, writing – review & editing. **Hannah Mayjor:** conceptualization, data curation, writing – review and editing. **Portia Freeman:** conceptualization, writing – review and editing, data curation. **Fiona Rees:** writing – review and editing, data curation, resources. **Renee Lovell:** writing – review and editing, resources.

## Conflicts of Interest

The authors declare no conflicts of interest.

## Data Availability

The data that supported the findings of the study are available from the corresponding author upon reasonable request.
